# Lectures on the Diagnosis and Treatment of Diseases of the Lungs

**Published:** 1840-11-28

**Authors:** W. W. Gerhard


### MEDICAL EXAMINER.

DEVOTED TO MEDICINE, SURGERY, AND THE COLLATERAL SCIENCES.

No. 48.] PHILADELPHIA, SATURDAY, NOVEMBER 28, 1840. [Vol. III.

### LECTURES ON THE DIAGNOSIS AND TREATMENT OF DISEASES OF THE LUNGS.

BY W. W. GERHARD, M. D.

### LECTURE XI.—(*Continued.*)

PNEUMONIA.

*Treatment.*—The treatment of frank pneumonia is that of ordinary inflammation modified by the peculiarities of the organ affected. Hence bleeding is the most efficient remedy, and should be practised freely at the beginning of the disorder. The method which has of late years been revived by Dr. Bouillaud, consists in repeated bleedings, which are prescribed again and again for several days. This method is reduced to a regular formula, and in the hospital practice there are not so many obstacles to this system as in private; but it must be obvious to every one that no one method of treatment, or at least no regular formula, is applicable to all cases, and I do not, therefore, advise a uniform method of blood-letting. The best directions must be gathered from a knowledge of the disorder, and from the present symptoms of the patient. Thus, in the commencement, a very large bleeding, pushed to the verge of syncope, is certainly best in a plethoric individual, or a moderately strong person, previously in good health, if the pneumonia is of a highly inflammatory kind—that is, if the evidence of vascular excitement be decided; for it is in these cases that the inflammation tends necessarily to diffuse itself, as it were, over a large surface, and to attack several organs, especially the serous tissues of the circulatory system. A large bleeding is of course the surest means of checking this tendency, and is the most comforting remedy for the patient, as it at once diminishes the headach and the oppression which are amongst the most disagreeable symptoms. A general bleeding produces much more effect than a local one, which is almost nugatory in its action upon the highly inflammatory cases of pneumonia, although very powerful in the later stages, or in the slighter forms of the disorder. The venesection may be repeated on several successive days, or in the after-part of the days in which the first bleeding was practised, if it seem necessary from the excitement of the pulse and the vascular action. That is, if the pulse should rise again, and especially if it should become more developed after the first bleeding. It is, as you may readily suppose, impossible to lay down positive and unvarying directions for conditions of things which are in their nature changeable. But by reflecting on the condition of the lung, which at first is merely that of engorgement or commencing hepatization, and on the stimulant properties of the inflammatory blood, it is easy to see that several bleedings may become necessary, although in the majority of cases one single bleeding will suffice, especially if the sedative effects of it be kept up by other remedies, particularly the antimonials.

The appearance of the blood drawn, is, of course, highly inflammatory,—that is, much buffed, with a very firm crassamentum; this is a tolerably correct, but not a sure guide for the repetition of venesection. The blood will generally remain buffed, even in that period of the disease when bleeding is no longer of benefit.

A physician is frequently called to a patient late in the disorder, when the inflammation has either not been treated by bleeding, or the disease continues very severe. It is very difficult in these cases to decide as to the propriety of general bleeding; my own impression is, that bleeding is in these cases apt to produce a double influence, which is partly of mischief, and partly of benefit. The inflammation, which is generally commencing in certain parts of the lung, or at least is much less advanced than in others, may be, to some degree, checked by the blood-letting; but those portions of the tissue in which the blood is completely stagnant, and, as it were, incorporated with it, are restored with more difficulty if blood be drawn from the general system. This is still more strongly the case, if the pneumonia has passed into the third or suppurative stage. The effect of blood-letting upon the general circulation is also in these cases often productive of evil, for the coagula which begin to form in the heart may become a greater obstacle to the circulation if the strength of the patient be lessened. The latter effect is difficult to demonstrate; but it has struck me in a number of cases that it was founded on good grounds, and I therefore state it for what it may be worth.

The action of local depletory means in acute sthenic pneumonia is much more limited than that of general bleeding; the beneficial effects of these remedies is almost confined to the latter stages of the disorder, when a portion of the lung remains in the first or second stage of the inflammation, but the greater part of it has passed into the third stage. The local bleeding, then, seems to get rid of the remaining inflammation with less exhaustion of strength. When we meet with patients who have been neglected during the greater part of an attack of pneumonia, we are often obliged to limit our depletory measures to cups or leeches.

Blisters, or tartar emetic ointment, are not necessary as a general rule in acute pneumonia; for the disease belongs to those inflammatory disorders, for the earlier stages of which blisters are not adapted. They are useful, however, at the beginning of the third stage, when the benefit from them is scarcely equalled by that from any other remedy in the treatment of pneumonia. The blisters then act with great power in checking the inflammation, at the same time preventing the collapse which is so frequent at this stage of the disease. The blister should be rather large, and in general the best place for it is under the axilla, or between the scapula and the spine. Tartar emetic ointment applied so as to produce a very rapid pustulation, has been recommended under similar circumstances; but I do not in general regard it as possessing any advantages over blisters, while it is for many reasons inconvenient. Sinapisms, or other rubefacients, are often useful within certain limits,—that is, as stimulants to the general strength, and as remedies which have a powerful influence upon the dyspnœa which attends the disorder.

Next in importance to general blood-letting as a remedy in pneumonia, is the tartrate of antimony; this medicine may be given in several ways, either as a simple diaphoretic expectorant, or as a direct arterial sedative. In the former case it should be given in doses from a twelfth to a quarter of a grain every two hours; in general a sixth of a grain is borne at first, and afterwards the patient should take a quarter of a grain, either alone, or combined with nitre or calomel. The medicine is, in these doses, quite free from danger, except in a very few individuals of extremely irritable temperament; for there are some patients who cannot bear antimony in any dose, or in any form. In most cases, however, these small doses of a sixth of a grain are attended with a disposition to sweating, and a diminution in the excitement of the circulation, which, on the other hand, always coincides with a diminution of the general inflammatory action, which plays so important a part in the pathology of acute pneumonia.

Of late years the contra-stimulant, or Italian method of giving antimony in very large doses, has been much resorted to in the treatment of pneumonia. This method has been perfected in France, and rendered much more safe. In my own practice I have adopted very nearly the usual formula of the French hospitals; it is as follows: Tart. Antim. gr. vj.; Aq. Menthæ. ℥vj.; Gum. Acac. ʒij. *M.* Of this a tablespoonful may be taken every two hours. It is not always customary to add the gum arabic, but the irritation of the stomach is certainly lessened by it. The antimony, taken in this dose, rarely produces any other effect than purging, which does not invariably follow. If the purging is severe, it is readily checked by adding a few drops of laudanum to each dose. In itself opium is objectionable, but it may be properly used if there is a decided tendency to purging.

The medicine should be continued in this dose for twenty-four hours, and not increased until the next day, when eight grains may be given instead of six; either in the same or in a larger quantity of vehicle; it is better to avoid giving it in too concentrated a form. If the tolerance has been established the first day,—that is, if the remedy has not produced decided puking or purging, or very debilitating sweats, it may be safely taken during the second day; and if the disease does not abate, the dose for the third day should be the same as that for the second. But, after the third day, there is some danger in continuing the antimony in a high dose, unless the patient is perfectly conscious, and his brain entirely clear; if the remedy is then attended by no uncomfortable sensations, there is little danger in its administration. But if the patient is comatose, or even slightly stupid, very extensive inflammation and other structural lesions may follow the tartarized antimony without any symptoms to indicate them. If the cerebral functions are unimpaired, the condition of the nervous system is a very faithful guide for the administration of the tartarized antimony. The good effects of the medicine are shown by the diminution of the local signs, and of the oppression and fever; this is especially obvious in the local signs of the pulmonary inflammation, for the antimony seems to act more quickly upon the parenchyma of the lungs than even general bleeding. When the symptoms have declined, the remedy should be gradually diminished, and not suddenly discontinued; about two grains should be taken from the dose, each day, until the whole amount is withdrawn.

There is some danger in attempting to give antimony in these doses to certain individuals who possess a peculiar idiosyncrasy with regard to the medicine; for there are some persons who cannot bear it in any form, or even in small doses, without great nervous distress and extreme prostration: to such persons the remedy should never be given, at least not in any other than in very minute doses. Besides these peculiar cases, the antimony will occasionally produce injurious effects from the mere purging or excessive emesis which it ocsions,—chiefly from the former cause. It is true that the addition of a small quantity of opium, or even the mere persistence in the remedy, will often suffice to arrest such a tendency; but if the patient should not lose this extreme susceptibility, it becomes necessary to discontinue the antimony.

The remedy which is next in power to antimony is mercury, although its effects are somewhat different. When given in the period of hepatization it acts in two ways,—as a directly antiphlogistic remedy, and as possessing a peculiar power in preventing the formation of lymph,—in other words, it is antiplastic. Hence, when given after bleeding, it is directly opposed to the progress of the inflammation, and modifies the products which result from it. Mercury should be given in such doses as to produce a full impression upon the general system, not amounting to ptyalism, but producing a slight action upon the gums, as an evidence of its constitutional effect. The proper dose is from a quarter to half a grain of calomel every two hours if it be desirable to make a rapid impression; from a third to half a grain three times daily if the mercury be designed to act more slowly. Even less doses produce at times a good effect. The calomel is often combined with ipecacuanha or opium; but the latter remedy should be given with great reserve in acute inflammatory pneumonia: the ipecacuanha is free from danger, and is generally of service by its power of facilitating the operation.

The mercurial impression is generally followed by a rapid decline of both general and local symptoms. If it should fail, the disease assumes one of two forms,—it either remains in the highly inflammatory condition, or it passes into the third stage of the disease. In the first case it may become necessary to recur again to depletory measures; in the second, blisters to the chest, with stimulating expectorants, and sometimes wine whey, or in persons addicted to the abuse of alcohol, milk punch may become necessary.

The expectorants which are of most value when the antiphlogistic treatment has failed, are the eupatorium and senega, or the sanguinaria. These may be given in the form of infusion—half an ounce of eupatorium, and two drachms of senega in a pint of boiling water—of which from a table-spoonful to a wine-glassful, according to the susceptibility of the patient, should be taken every two or three hours; or the senega and sanguinaria may be combined in the dose of two or three drachms of senega, and one of sanguinaria, or half a pint of boiling water, and a table-spoonful given every two hours, unless it should excite much nausea. In a few cases the dose may be increased.

After the acute symptoms of pneumonia are dissipated, the patient will often continue to cough a little; and on examination it will be found that the bronchial respiration has not entirely ceased at the root of the lung. This state of things depends upon the very slow absorption of the substance which is effused into the cellular tissue of the lung. It requires no special treatment, and in a little while will cease; still the patient should avoid exposure, and to aid in this object he may wear a Burgundy pitch-plaster, or some similar covering over the part affected.

### LOCAL PNEUMONIA.

Besides the highly inflammatory cases of pneumonia, there is a variety of the disorder which is simple and inflammatory, but local, attacking only a small portion of the lung, and therefore not producing the general inflammatory action of the severer cases. The local signs of pneumonia are present in these cases, such as bronchial respiration and crepitant rhonchus; but the fever is moderate, or may not exist at all, and the prostration is but slight. These cases cannot be distinguished from ordinary catarrh, except by the local signs and the expectoration, which is generally, but not invariably, characteristic.

The duration of these cases rarely exceeds a fortnight, but in general it does not extend beyond ten or twelve days. The patient is not often confined to his bed, and in some cases he feels so little inconvenience that he will insist upon going out and following his usual employments. The prognosis is always favourable, unless some unexpected aggravation of the disease should take place.

The treatment in this form of local pneumonia is extremely simple. The disease tends so universally to recovery that there is little difficulty in its management, and the large majority of cases would get well under any treatment. It is, however, quite possible to hasten its course. For this purpose the best remedies are, at the very commencement, a moderate bleeding, or after the first few days one or two applications of cups to the affected part. These remedies will relieve the lung, and facilitate the cure, which is brought about by exciting secretion from the inflamed surface. If the secretion takes place readily, or if the inflammation is very slight, blood-letting in every form is not necessary; but if the pulse be at all excited, the symptoms are more or less relieved by it, and no inconvenience at least will result. Bleeding, however, is never followed by the same decided benefit as in the cases of highly inflammatory pneumonia.

The secretions from the lungs are promoted by the same treatment which is applicable to the declining stages of the last mentioned variety,—that is, the infusion or syrup of senega or ipecacuanha, or infusion of eupatorium or sanguinaria, or combinations of these with the senega. Small doses of the antimonials are also productive of prompt relief when the patient is feverish, but I do not regard the antimonials as so generally useful or safe as the vegetable expectorants. Towards the decline of local pneumonia the case requires some attention to distinguish between those cases which are really simple, and those in which there is a complication of pulmonary tubercles: in the latter case the disorder may pass into phthisis; in fact, it is then only one mode of attack of the latter disease.

